# Prediction of Clinical Factors Associated with Pandemic Influenza A (H1N1) 2009 in Pakistan

**DOI:** 10.1371/journal.pone.0089178

**Published:** 2014-02-24

**Authors:** Nadia Nisar, Uzma Bashir Aamir, Nazish Badar, Muhammad Rashid Mehmood, Muhammad Masroor Alam, Birjees Mazher Kazi, Syed Sohail Zahoor Zaidi

**Affiliations:** Department of Virology, National Institute of Health, Chak Shahzad, Islamabad, Pakistan; University Hospital San Giovanni Battista di Torino, Italy

## Abstract

**Background:**

Influenza is a viral infection that can lead to serious complications and death(s) in vulnerable groups if not diagnosed and managed in a timely manner. This study was conducted to improve the accuracy of predicting influenza through various clinical and statistical models.

**Methodology:**

A retrospective cross sectional analysis was done on demographic and epidemiological data collected from March 2009 to March 2010. Patients were classified as ILI or SARI using WHO case definitions. Respiratory specimens were tested by RT-PCR. Clinical symptoms and co-morbid conditions were analyzed using binary logistic regression models.

**Results:**

In the first approach, analysis compared children (≤12) and adults (>12). Of 1,243 cases, 262 (21%) tested positive for A(H1N1)pdm09 and the proportion of children (≤12) and adults (>12) were 27% and 73% respectively. Four symptoms predicted influenza in children: fever (OR 2.849, 95% CI 1.931–8.722), cough (OR 1.99, 95% CI 1.512–3.643), diarrhea (OR 2.100, 95% CI 2.040–3.25) and respiratory disease (OR 3.269, 95% CI 2.128–12.624). In adults, the strongest clinical predictor was fever (OR 2.80, 95% CI 1.025–3.135) followed by cough (OR 1.431, 95% CI 1.032–2.815). In the second instance, patients were separated into two groups: SARI 326 (26%) and ILI 917 (74%) cases. Male to female ratio was 1.41∶1.12 for SARI and 2∶1.5 for ILI cases. Chi-square test showed that fever, cough and sore throat were significant factors for A(H1N1)pdm09 infections (*p* = 0.008).

**Conclusion:**

Studies in a primary care setting should be encouraged focused on patients with influenza-like illness to develop sensitive clinical case definition that will help to improve accuracy of detecting influenza infections. Formulation of a standard “one size fits all” case definition that best correlates with influenza infections can help guide decisions for additional diagnostic testing and also discourage unjustified antibiotic prescription and usage in clinical practice.

## Introduction

Influenza has been recognized and documented as a human respiratory disease over 2000 years [1]. The causative agents are influenza viruses types A, B and rarely C, which circulate continually among human population throughout the world [2]. In early 2009, influenza A/H1N1 emerged as a global pandemic threat with over 15000 reported deaths, from more than 209 countries [3]–[5]. The highest attack rates were reported amongst young adults as compared to other age groups, a pattern similar to the 1918–1919 H1N1 pandemic during which nearly half of the influenza-related deaths occurred among young (20–40 years) and previously healthy adults [6].

The precipitous spread of A(H1N1)pdm09 virus highlighted yet again the need for availability of appropriate and prompt diagnostic tools with equivalent emphasis on both clinical and laboratory facilities to prevent disease transmission and institute successful treatment. Even though several laboratory tests, including real-time reverse transcriptase-polymerase chain reaction (RT-PCR), became available quite rapidly to provide confirmatory diagnosis for A(H1N1)pdm09 but laboratory tests were not always accessible during outbreaks and clinical judgment of attending physicians became a major factor in the timely identification of new influenza cases. This highlights the necessity to determine clinical predictors of influenza infection for diagnosis in patients presenting with respiratory illness, in order to direct appropriate antiviral therapy, and to prevent unwarranted antibiotic use.

Symptomatic predictors of the etiology of infectious diseases are necessary when swift response is required for management and treatment in a pandemic setting [7]. The nonspecific presentation of influenza infection makes it difficult to distinguish from other febrile or respiratory illnesses. The data on clinical predictors of influenza in pediatric populations are scarce [1]. Several studies have highlighted differences in clinical predictors of influenza in adults and children with influenza-like illness [1]–[2]. In contrast to adults, children play a primary role in transmission of seasonal influenza, due to their increased susceptibility to influenza infections and prolonged viral shedding. Evidence shows that influenza often spreads earliest among school-age children and these children are considered as appropriate sentinels for the beginning of influenza circulation in the community and contribute towards its spread more than adults [2], [4], [8].

Numerous aspects of epidemiological characteristics, clinical manifestations, and outcomes of the recent pandemic have not been fully understood. This study evaluated the key clinical predictors for this novel strain in children and adults and compared different clinical diagnostic criteria with laboratory diagnostic tests. A comparative analysis of A(H1N1)pdm09 infections was carried out based on both outpatients with influenza like-illness (ILI) and hospitalized cases with severe acute respiratory illness (SARI) to improve the accuracy of predicting an influenza infection.

## Materials and Methods

### Data Collection

Demographic and epidemiological data was collected from all suspected cases that reported at designated sentinel sites across the country and outbreak cases were referred through Epidemic Investigation Cell (EIC) at NIH, Islamabad. A retrospective cross-sectional analysis was done using non-probability sampling from March 2009 to March 2010 during the A(H1N1)pdm09 period. A total of 1243 cases were included in this analysis. All cases with no clinical information or those that did not meet inclusion criteria (case definitions for ILI & SARI) were excluded from the study. The data was recorded at the time of sample collection. Two type of analysis were carried out. In order to reduce recall bias, the demographic and clinical information was counter checked from the hospital records following informed consent. The study was approved by the internal review committee of the National Institute of Health-Pakistan. A written consent was obtained from each subject through their parents and/or guardian by marking a check box as given on the patient data collection form to document the consent taking procedure; however, the patient identities were not disclosed at any stage. Also, a verbal consent was taken from few subjects as the study setting involved sampling of suspected pandemic cases. The institutional committee was informed of the specific needs with reference to study setting and approved this mode of consent.

### Case Definition

The cases selected for influenza testing were screened as either influenza-like illness (ILI) or Severe Acute Respiratory Illness (SARI). The standard WHO case definitions were used; ILI case was defined as a person with Acute Respiratory Illness, measured temperature of ≥38°C and cough within past seven days of onset. The SARI cases were defined as those with Acute Respiratory Illness, measured fever of ≥38°C, cough within past seven days of onset and requiring hospital admission [9].

### Study Settings and Design

In first approach, the cases were separated into two groups on the basis of age i.e., children (≤12 years) and adults (>12 years). Secondly, patients were evaluated under ILI and SARI groups based on WHO case definitions. The cases selected for influenza testing were screened as either influenza-like illness (ILI) or Severe Acute Respiratory Illness (SARI) that were enrolled in the surveillance system by sensitized and trained hospital physicians using a standard case report form.

### Laboratory Procedure

According to WHO guidelines; nasopharyngeal or throat swabs were collected from all patients and transported to WHO National Influenza center at the Department of Virology, National Institute of Health-Islamabad. Samples were tested for A(H1N1)pdm09 using the CDC Real time RT-PCR protocol for pandemic H1N1 and positive samples were characterized using methodologies as described previously [9].

### Statistical Analyses

The analysis was carried out on children (≤12) and adults separately/independently. Data on gender, age and clinical symptoms was analyzed for patients of influenza like illness (ILI) and severe acute respiratory illness (SARI). Various parameters were compared between influenza positive and negative cases. The aim of the statistical analysis was to find the best clinical predictors of influenza infection for children and adults. Student’s test was used for comparison of continuous variables; while Fisher’s exact test was used for comparison of dichotomous variables. Logistic regression analysis {along with calculation of odds ratio (OR) and 95% confidence interval (CI)} was performed to determine the best clinical predictors {fever, dry cough, sore throat, shortness of breath and diarrhea along with co-morbid conditions (such as cardiac, hepatic, respiratory and metabolic diseases)} for influenza virus infection. Positive Predictive values (PPV) were calculated for each symptom and combination of symptoms in logistic regression analysis, independently or in combination with other symptoms. A *p-*value less then 0.05 (two-sided) was considered to be statistically significant. SPSS version 16 was used for statistical analysis.

## Results

**Figure 1:**
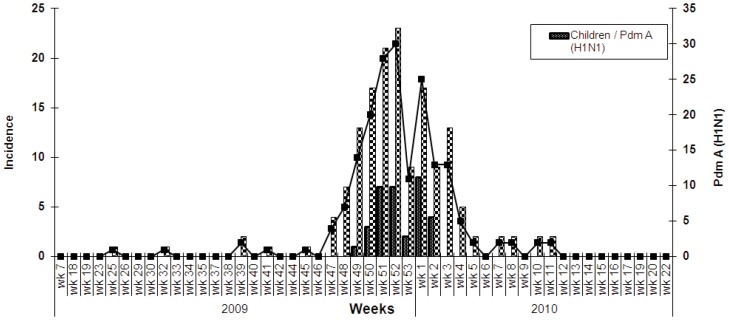
Incidence of pandemic Influenza A(H1N1)2009 among children and Adults.

During the study period 1243 specimens were tested by Real Time RT-PCR; 262 (21%) were positive for influenza A(H1N1)pdm09. The peak influenza activity was found in week 52 of year 2009 (17%). The incidence of influenza positive cases in adults and children was high in week 52 of year 2009 and week 1 of year 2010, respectively (Figure 1). Mean age of Influenza positive and negative cases was 29.95±16.95 and 32.90±19.47 years, respectively. Independent sample t-test showed that the mean age of A(H1N1)pdm09 negative cases was significantly higher as compared to the positive cases (*p* = 0.034).

Furthermore, higher positivity rate was observed for adults (73%) as compared to children (27%). Mean age for children was 5.15±3.60 years and 36.96±16.66 years in adults. Gender ratios M: F amongst children and adults were 1.36∶1.03 and 1.78∶1.34 respectively. The predictive clinical signs and symptoms distinctive to influenza illness detection between SARI and ILI groups were analyzed. Independent sample t-test showed that there was a significant age difference between ILI and SARI patients (*p* = 0.001), and mean age of SARI patients was higher (41±20.57) than ILI cases (28.85±17.24). The percentage of A(H1N1)pdm09 positive cases was higher for ILI than SARI and significant association was determined on chi-square test application (*p* = 0.008) (Table 1).

**Table 1:** Comparative analysis of demographics and epidemiological factors between children and adults.

Groups	No. (%)	Age (in Years) Mean ± S.D	Gender, M:F Number (%)	Influenza positive, No. (%)
Children (≤12)	242 (19)	5.15±3.60	87(57): 66(43)^#^	70 (27)
Adult (>12)	1001 (81)	36.96±16.66	519(57): 389 (43)^$^	192 (73)
*p*-value		0.034*	0.520	0.008*
**Presentation Status**				
SARI Patients	326 (27)	41±20.57	161(53): 143(47)	88(27)
ILI Patients	917 (73)	28.85±17.24	395(52): 362 (48)	174(19)
*p*-value	0.008*	0.001*	0.550	0.009*

* = Significant; M = Male; F = Female.

# = gender data was available for 153 out of 242 in children group.

**$ = **gender data was available for 908 out of 1001 in adult group.

Logistic regression analysis in children showed that fever ≥38°C (odds ratio [OR] 2.849, 95% confidence interval [CI] 1.931–8.722), cough (OR 1.99, 95% CI 1.512–3.643), diarrhea (OR 2.100, 95% CI 2.040–3.25) and respiratory disease (OR 3.269, 95% CI 2.128–12.624) had significant association with influenza positivity i.e. detection of A(H1N1)pdm09 (*p*<0.05). However sore throat, shortness of breath, allergies, heart, liver and metabolic diseases were less common in children. On the other hand, in adults group, fever ≥38°C (OR 2.80 95% CI 1.025–3.135), cough (OR 1.431, 95% CI 1.032–2.815) and smoking (OR 1.978, 95% CI 1.959–2.315) were significantly related to influenza infection (*p*<0.05) (Table 2).

**Table 2:** Binary logistic regression of clinical symptoms and underlying medical conditions of Influenza positive children and adults.

Characteristic	Children			Adult		
Symptoms and signs	OR^1^	95% CI^2^	*p*-value	OR^1^	95% CI^2^	*p*-value
Fever	2.849	1.931–8.722	0.048*	2.80	1.025–3.135	0.038*
Cough	1.99	1.512–3.643	0.032*	1.431	1.032–2.815	0.033*
Sore throat	1.310	0.556–3.088	0.537	1.317	0.923–1.878	0.128
Shortness of breath	1.171	0.522–2.628	0.702	1.187	0.824–1.710	0.357
Diarrhea	2.100	2.040–3.25	0.040*	1.033	0.929–1.798	0.646
Allergies	0.748	0.084–6.642	0.794	1.457	0.728–2.919	0.285
Respiratory Disease	3.269	2.128–12.624	0.041*	1.453	0.810–2.608	0.208
Liver Disease	3.871	0.235–16.64	0.309	0.984	0.281–3.441	0.980
Metabolic Disease	3.871	0.235–18.64	0.309	1.072	0.401–2.866	0.889
Cardiac Disease	1.969	1.910–2.031	0.839	1.009	0.969–1.052	0.675
Smoking	–	–	–	1.978	1.959–2.315	0.044*

OR^1^ = odds ratio; CI^2^ = Confidence Interval; * = Significant.

Of the 1243 enrolled patients, 262 (21%) had laboratory-confirmed influenza, of whom, 88 (34%) were SARI, 174 (66%) were ILI and the proportion of laboratory-confirmed influenza A(H1N1)pdm09 cases was highest in age group between 20–40 years of age (70/160; 44% in SARI, 104/401; 26% in ILI,). Among the SARI patients, 34% had one or more underlying medical condition placing them at a high risk for influenza-related complications. The most commonly reported clinical symptoms among A(H1N1)pdm09 related hospitalized patients were fever (72%), cough (82%), sore throat (63%), shortness of breath (56%) and diarrhea (15%). In comparison with ILI patients, the incidence of underlying medical conditions was higher in hospitalized patients as allergies identified in (6%), respiratory disease in (18%), liver disease in (4%), metabolic disease in (7%) and cardiac disease in (13%) cases (Table 3).

**Table 3:** Clinical Symptoms and Underlying Medical Conditions among the Hospitalized (SARI) and Non-Hospitalized (ILI) Cases.

Characteristics	All Patients (n = 1243) n (%)	SARI Patients (n = 326) n (%)	ILI Patients (n = 917) n (%)
**Symptoms**			
Fever	699(56)	234(72)	465(51)
Cough	730(59)	267(82)	463(51)
Sore throat	653(53)	205(63)	448(49)
Shortness of breath	354(29)	183(56)	171(19)
Diarrhea	78(6)	48(15)	30(03)
**Underlying Medical Conditions**	262(27)	88(27)	174(66)
Allergies	57(5)	20(6)	37(2)
Respiratory Disease	77(6.2)	58(18)	19(2)
Liver Disease	20(2)	13(4)	7(1)
Metabolic Disease	30(2.4)	22(7)	8(1)
Cardiac Disease	56(5)	43(13)	13(1.4)

SARI = Severe Acute respiratory infections; ILI = Influenza like Illness.

All fatal cases of influenza A(H1N1)pdm09 (n = 29) were adults with mean age of 39±14 years. The distribution of fatal cases was higher among SARI patients (n = 19, 65.5%) as compared to ILI (n = 10, 34.5%).

## Discussion

Pandemics behave as unpredictably as the viruses that cause them. Apart from the inherent lethality of the virus, its capacity to cause severe disease in non-traditional age groups, namely young adults, is a major determinant of a pandemic’s overall impact. During the previous century novel influenza virus with pandemic influenza potential demonstrated highly variable pattern of spread, disease severity and mortality rates. Early and accurate diagnosis in potential pandemic situation can help ensure prompt and appropriate treatment that ultimately decreases the economic and public health burden [10].

Although the clinical features and course of illness in patients during influenza outbreaks have been previously described, data comparing disparities in clinical presentations between A(H1N1)pdm09, seasonal influenza and ARIs is quite limiting [6]. The unique genetic and antigenic features of variant influenza strains resulted in a dramatically rapid global spread since it first appeared in April 2009 but most illnesses were acute and self-limiting, with the highest attack rates among children and young adults [7]. The purpose of this study was to define better clinical predictors for Influenza among A(H1N1)pdm09 cases on the basis of age groups and secondly, by case definitions of outpatient (ILI) and inpatient (SARI) groups.

During the A(H1N1)pdm09 pandemic, young adults and middle age individuals had higher infection rates as compared to other age groups [3], [6]. Conversely, during seasonal epidemics influenza mortality is classically highest in the elderly population [3]. This shift of mortality toward younger age group is a signature feature of pandemics and is particularly important as young adults constitute productive age group and therefore early intervention and treatment may reduce economical losses substantially [5]. This age based data can help public health authorities to better organize appropriate response strategies to prevent future pandemics.

In this study we also compared clinical features between the inpatients presenting with SARI (Severe Acute Respiratory Illness) and outpatients or ILI (Influenza Like Illness) cases. In general, hospitalized cases are more seriously ill or had predisposing conditions leading to a complicated course of illness. Adults between 20 to 40 years age accounted for over 65% of laboratory-confirmed H1N1 pandemic influenza infections among hospitalized SARI cases. In contrast other studies have documented higher hospitalization rates in children as compared to adults [11]–[13]. Elderly (>60 yrs) are more prone to become severely ill with seasonal influenza, however, it was observed during pandemic that elderly cases were relatively spared, possibly due to previous exposure to H1N1 viruses and resulting immunological memory [14].

It is a well-established fact that the sensitivity of clinical predictors for influenza varies depending on a multitude of factors including prevalence of disease, age, underlying illnesses, duration of symptoms prior to consultation and the vaccination status in the population being evaluated. Published data to date has shown varying positive predictive values when using fever and cough as clinical predictors [15]–[16]. Numerous studies on predictive symptoms for influenza infections have included children but did not analyze them as a distinct group from adults [17]–[18]. Our study showed that A(H1N1)pdm09 cases were more likely to present with fever and cough in both children as well as adults with positive predictive values up to (PPV) 69.1% and 70.4% respectively but were less liable to have sore throat and shortness of breath. In congress to our findings, many other studies have reported that fever and cough increased the probability of infection with A(H1N1)pdm09 in all age groups [19]–[23]. Similarly, Monto et al. and Michiels et. al. have reported fever and cough as best predictors with substantially high PPVs (86.8% and 79%) and suggested that physicians could correctly diagnose influenza in over 60–70% of their patients on the basis of clinical symptoms alone [8], [15]–[17].

In our study we found that in children along with fever and cough addition of diarrhea increased the PPV up to 73.3%. In contrast, Chen-Yen Kuo et al., have reported diarrhea as an insignificant symptom for pandemic influenza [16]. Even though gastrointestinal symptoms are not reported often for human influenza, almost all avian and some swine viruses are secreted through the intestinal tract. It stands to reason that gastrointestinal symptoms such as diarrhea might be an important influenza-specific clinical trait [20].

Currently, definition of influenza-like illness varies considerably from country to country, Most definitions of Influenza-like illness include fever, feverishness, myalgia, general weakness, headache and respiratory symptoms [21], [22]. This symptom complex overlaps amongst various respiratory pathogens in addition to influenza such as adenovirus, parainfluenza virus, respiratory syncytial virus or enteroviruses and may be difficult to differentiate clinically alone. The CDC and WHO ILI criteria used during 2009 pandemic were specific enough to differentiate suspected cases but were not sensitive enough to detect all cases. In daily practice, it is impractical, expensive and time consuming to swab and test all patients with acute respiratory symptoms suggestive of an influenza infection [19]–[21], [23]. Implementation of sentinel surveillance systems to detect the influenza virus in the community through a combination of epidemiological, clinical and virological information can help to evaluate these predictive tools in local and regional settings.

There are some limitations to our study. Our analysis was conducted using 12 years age cut off which is different from age based reports by other groups [1], [21] and may not be comparable. As we did not analyze other subtypes of influenza viruses, nor did we analyze non-viral respiratory pathogens, arguably the proposed clinical predictors/criteria may be relevant only to A(H1N1)pdm09 infections and not applicable to pandemics with other influenza strains and respiratory pathogens. Secondly, the retrospective nature of our data analysis limits it utility.

Future studies in outpatient setting should be encouraged including patients with influenza-like illness to develop sensitive clinical case definitions which strengthen the identification of Influenza cases and can improve the accuracy of predicting influenza infection. Formulation of a standardized group of array that best correlates with influenza infections can be quite helpful to guide decisions for further diagnostic testing and discourage unjustified antibiotic prescription in clinical practice.
